# SWARAM: Osprey Optimization Algorithm-Based Energy-Efficient Cluster Head Selection for Wireless Sensor Network-Based Internet of Things

**DOI:** 10.3390/s24020521

**Published:** 2024-01-14

**Authors:** Ramasubbareddy Somula, Yongyun Cho, Bhabendu Kumar Mohanta

**Affiliations:** 1 Department of Information and Communication Engineering, Sunchon National University, Suncheon-si 57922, Republic of Korea; subbu1219@scnu.ac.kr; 2 Department of CSE, Koneru Lakshmaiah Education Foundation, Vaddeswaram 520002, Andhra Pradesh, India

**Keywords:** Internet of Things, wireless sensor network, clustering protocol, energy conservation, osprey optimization algorithm

## Abstract

The Internet of Things (IoT) has transformed various aspects of human life nowadays. In the IoT transformative paradigm, sensor nodes are enabled to connect multiple physical devices and systems over the network to collect data from remote places, namely, precision agriculture, wildlife conservation, intelligent forestry, and so on. The battery life of sensor nodes is limited, affecting the network’s lifetime, and requires continuous maintenance. Energy conservation has become a severe problem of IoT. Clustering is essential in IoT to optimize energy efficiency and network longevity. In recent years, many clustering protocols have been proposed to improve network lifetime by conserving energy. However, the network experiences an energy-hole issue due to picking an inappropriate Cluster Head (CH). CH node is designated to manage and coordinate communication among nodes in a particular cluster. The redundant data transmission is avoided to conserve energy by collecting and aggregating from other nodes in clusters. CH plays a pivotal role in achieving efficient energy optimization and network performance. To address this problem, we have proposed an osprey optimization algorithm based on energy-efficient cluster head selection (SWARAM) in a wireless sensor network-based Internet of Things to pick the best CH in the cluster. The proposed SWARAM approach consists of two phases, namely, cluster formation and CH selection. The nodes are clustered using Euclidean distance before the CH node is selected using the SWARAM technique. Simulation of the proposed SWARAM algorithm is carried out in the MATLAB2019a tool. The performance of the SWARAM algorithm compared with existing EECHS-ARO, HSWO, and EECHIGWO CH selection algorithms. The suggested SWARAM improves packet delivery ratio and network lifetime by 10% and 10%, respectively. Consequently, the overall performance of the network is improved.

## 1. Introduction

The Internet of Things (IoT) has become an emerging paradigm research area in academia and industries. Human life is significantly impacted in many aspects by IoT in terms of convenience, enhanced experiences, and efficiency. The IoT concept was introduced by Kevin Ashton in 1999 [1,2,3]. Many physical objects in IoT are embedded with computing capabilities, allowing them to communicate with other devices to exchange collected data seamlessly and automatically. IoT real-time applications include smart homes, healthcare, urban living, retail and commerce, agriculture, transportation, and country border surveillance [4,5,6]. The IoT enhances the efficiency of complex systems by using data and connectivity.

The wireless sensor networks (WSNs) consist of distributed sensor nodes capable of collecting data about surrounding environment conditions and sending it to the destination node over the network for analysis. The sensors collected data forward to preprocessing via an electrical signal. The communication element is vital in transmitting and receiving data wirelessly in WSN [7,8,9]. Small sensors with low power consumption are now available due to advanced technologies. WSN real-time applications are spread across a multitude of industries, such as intelligent infrastructure, military systems, and industrial automation. In a few cases, the WSN requires many sensor nodes to handle real-time applications. WSN can dynamically change the network structure due to various external events, such as node mobility, environmental changes, and the adding or removing of nodes to optimize the effectiveness of the network [10,11,12].

The sensor nodes are furnished with limited processing power, low memory capacity, and little battery power, so utilizing resource-constrained sensor nodes to improve the overall network lifetime is a daunting challenge in WSN. Different approaches have been proposed to enhance the lifetime of the network. Energy conservation during data transmission from source to destination is the main concern in WSN [13,14,15]. Routing is a precious commodity in WSN. The sensors’ count increases daily from a few to thousands of nodes, where sensor nodes relate to other sensors for multi-hop communication. The sensors are battery-powered, which is neither rechargeable nor replaceable. Existing works have adapted hierarchical routing, a clustering technique to handle energy issues in WSN. Therefore, clustering protocol is a significant approach to minimizing energy consumption and productively improving network lifetime.

Clustering is a popular approach for reducing energy consumption by minimizing redundant data transmission among the nodes. The sensor nodes in WSN are grouped into various clusters. In each cluster, the Cluster Head (CH) is allotted to communicate with other cluster members (CM). Many existing approaches proposed CH selection strategies by employing optimization algorithms. In the optimization algorithm, the optimal solution is selected among a set of solutions. Many optimization approaches have been used to pick up optimal CH in recent years, including particle swarm optimization (PSO) [16], artificial bee colony (ABC) [17], golden jackal optimization (GJO) [18], coati optimization algorithm (COA) [19], marine predator optimization (MPO) [20], and whale optimization algorithm [21]. However, the CH selection is performed with optimization algorithms that require considerable convergence time. As a result, sensor batteries are drained quickly. To solve this issue, we have contemplated utilizing the osprey optimization algorithm (OOA) to select the optimal CH in clusters to extend the network’s longevity. Compared to other optimization algorithms, the OOA convergence time is quick during the CH rotation process. This fast convergence plays a pivotal role in improving the overall performance of network and system adaptability. The following is the primary contribution of this work:

- First, the clusters are formed using distance parameters among nodes in the network. Later, CH is selected using the osprey optimization algorithm (OOA) in nodes.
- The CH selection model based on the osprey optimization algorithm is developed to increase network lifetime and throughput.
- The fitness function is formulated, including distance and residual energy parameters to obtain energy-efficient CH selection.
- The performance of the proposed SWARAM algorithm is simulated by conducting extensive simulation and compared with the performance of three benchmark CH selection algorithms, namely, EECHS-ARO, HSWO, and EECHIGWO.

This paper is divided into various sections as follows: firstly, a literature survey on different CH selection methods using multiple optimization algorithms is presented in Section 2. Then, the network model is discussed in Section 3. Next, CH selection using the proposed SWARAM approach is presented in Section 4. The performance of the proposed SWARAM is discussed in Section 5. Finally, Section 6 presents conclusions and future directions.

## 2. Related Works

In WSN, an energy-efficient strategy becomes a critical solution due to limited battery-powered sensor nodes deployed in inaccessible areas. The CH selection is an efficient technique to ensure network stability by forming clusters in the network area. Many designs are introduced to select CH, but enhancement of energy remains a challenging issue in WSNs.

Chaurasia et al. [22] suggested a metaheuristic-based cluster head section in WSNs (MOCRAW). It had two processes: optimal cluster head selection algorithm (CHSA) and Route Search Algorithm (RSA). In CHSA, the Energy Level Matrix (ELM) function was used to create the cluster. The ELM function chose CH based on distance, residual energy, density, and intercluster formation. The optimal path between source and destination was discovered by intercluster in RSA. In addition, the loop-free routing was aimed at achieving communication overhead. MOCRAM efficiency was accessed through various metrics, such as energy consumption, the number of living nodes, PDA, and latency. The simulation process was executed within the NS2 simulation environment. The overall energy efficiency of MOCRAM was improved by 8–10% compared to GAPSO-H, HMBCR, EAFTC-RIS, ECRP-UCA, and E-FUCA techniques.

Rami Reddy et al. [23] proposed the improved grey wolf optimization algorithm-based energy-efficient (EECHIGWO) technique to pick CH in WSNs. This technique aimed to choose optimal CH to improve network lifetime, network stability, energy efficiency, and average throughput in WSNs. In CH Selection, parameters such as average intracluster distance, residual energy, sink node distance, and CH node balancing factor were considered. The execution of the EECHIGWO technique was simulated in MATLAB and compared to state-of-the-art CH selection protocols FIGWO, FGWSTERP, HMGWO, LEACH-PRO, and SSMOECHS. The performance of the EECHIGWO scheme was tested in terms of energy consumption, number of dead nodes, average throughput, and operating rounds. The overall network stability of EECHIGWO was enhanced by 333.51%, 19.03%, 307.89%, 253.73%, and 169.29% compared with FIGWO, FGWSTERP, HMGWO, LEACH-PRO, and SSMOECHS in the network. However, the computation of CH selection by the fitness function requires more time.

Samiayya et al. [24] proposed hybrid snake whale optimization (HSWO) for optimal CH selection for enhancing network lifetime in WSNs. It had three phases: CH selection, initialization, and route selection. In CH selection, the optimal CH was selected considering various metrics such as distance and delay using the HSWO scheme. In initialization, the distance, energy, and population models were created. The sensed data were delivered to the destination via an optimal path without link breakages in route selection. The simulation was carried out using an NS2 simulation environment. The efficacy of the HSWO algorithm was compared with various performances of existing cluster head selection techniques, namely, ACI-GSO, PSO-EEC, ECRP-UCA, PSO-EEC, and BOA, to prove better results in WSNs. The network energy, throughput, energy consumption, packet delivery ratio, network lifetime, and computational time were achieved by 0.98, 0.975 Mbps, 0.59 mJ, 99.6%, 5600 rounds, and 0.1 s using the HSWO approach. However, The CH selection process takes more time.

Ramalingam et al. [25] proposed an artificial rabbits optimization-based energy-efficient algorithm (EECHS-ARO) to pick CH in WSNs. This approach aimed to improve network lifetime and reduce energy utilization in the network. To determine the optimal CH in the cluster, the fitness function was calculated utilizing remaining energy (RE), distance, and CH balancing factors. The simulation of the EECHS-ARO approach was evaluated in a MATLAB simulation environment. The performance of EECHS-ARO was evaluated compared to QOBOA, ALO, and TLBO. As a result, the packet delivery ratio and overall network lifetime were increased by 5% and 15%, respectively. However, it required more time to converge for CH selection in the network.

Arunachalam et al. [26] adapted the squirrel search algorithm (SSO-CHST) to extend the lifetime in the sensor network by using the gliding factor to ensure optimal cluster heads in WSN. The fitness function was computed to determine the CH and CM. The sensor node with a high fitness value was considered a CH, and the least value sensor nodes were considered CMs. The SSO-CHST technique was simulated using the MATLAB 2014a environment. The SSO-CHST method demonstrated a 15%, 12%, and 9% enhancement in network lifetime performance compared to the SFA-CHSS, ABC-CHSS, and ACO-CHSS benchmark methods. The SSO-CHST strategy demonstrated a 7%, 11%, and 14% reduction in energy consumption compared to the benchmark approaches SFA-CHSS, ABC-CHSS, and ACO-CHSS, respectively. The proposed SSO-CHST strategy decreased dead nodes by 13%, 10%, and 8% compared to the SFA-CHSS, ABC-CHSS, and ACO-CHSS approaches. However, it considered a number of simulation rounds to choose optimal CH.

Abraham et al. [27] proposed a Flamingo Search Algorithm-based energy-efficient CH selection in WSNs (FSA-CHS). The ultrascalable ensemble technique was adapted for clustering nodes and handling extensive data in the network. The shortest path was selected between BS and CH for transmitting data packets using the Q-learning approach to avoid issues due to complex network conditions. The fitness value was evaluated considering various parameters: energy consumption, distance between BS and CH, and coverage area. Experiments were conducted in MATLAB 2018a simulation environment. The performance of FSA-CHS in terms of total residual energy, alive nodes, time consumption, and count of dead nodes was contrasted with previous studies, including ACT, CI-ROA, Q-DAEER, and RL. The experiment results demonstrated that the FSA-CHS approach enhanced the network’s performance by reducing energy consumption and optimal path selection.

Ambareesh et al. [28] proposed Hybrid Jarratt Butterfly Optimization (HJBO) based on optimal route selection via CH. HJBO was merged with the FUZZY TOPSIS approach in optimal route selection to determine the optimal path. The aggregated data were transmitted to BS using ensemble clustering for minimum energy consumption. The performance of the HJBO approach was verified in terms of energy consumption, delay, throughput, and normalized overhead, network lifetime concerning the number of nodes in the MATLAB 2018a simulation environment. The results of the HJBO approach compared to existing systems such as E-ALWO, TBSEER, ER-SR, and EEGR. The network lifetime and throughput of HJBO were enhanced compared to benchmark works E-ALWO, TBSEER, ER-SR, and EEGR.

Jaya Pratha et al. [29] suggested hybrid CH selection in WSN to increase energy stability and network longevity. This paper proposed a hybrid Partha Mutualism Mechanism-inspired Butterfly and Flower Pollination Optimization Algorithm (HMMB-FPOA) to choose optimal CH based on multi-objectives, namely, residual energy, network cost, proximity, and network coverage. The overall convergence speed of the HMMB-FPOA algorithm exceeded the expected level due to the integration of FPOA and BOA algorithms. The HMMB-FPOA method was designed to achieve a balance between the exploitation and exploration stages, with the aim of extending the network’s overall lifetime. The performance of HMMB-FPOA was simulated in the MATLAB R2016a environment. The simulation results of HMMB-FPOA were compared with HIBSFL, HPSO-GA, HGWSOA, and CFFODE-SR CH selection schemes. HIBSFL, HPSO-GA, HGWSOA, and CFFODE-SR, with average throughput and residual energy, increased by 19.14% and 17.98%, respectively. The network lifetime and number of packets delivered to BS under different CHs were improved by 16.21% and 13.28% compared to benchmark CH selection schemes.

Sindhuja et al. [30] suggested energy-aware multi-object cluster head selection using African vulture optimization for secure data aggregation in WSN (MOCHSAPGAN-AVO). The MOCHSAPGAN-AVO performed a routing process through CH, and CH was selected based on fitness function using a self-attention-based progressive generative adversarial network. The fitness function was computed with various factors, including throughput, delay, energy, distance, and cluster density. The data was transferred to the base station via the optimal path once CH was selected. The optimal path was chosen based on three optimal parameters: connectivity, degree of satisfaction, and rate of service. The performance of MOCHSAPGAN-AVO was simulated in an NS2 simulation environment. The simulation results of MOCHSAPGAN-AVO compared with FL-CHESDA-WSN, DA-WSN-MLOA, and IDAF-ASLPPRR. The MOCHSAPGAN-AVO method demonstrated a 38.96%, 57.80%, and 41.97% lesser delay compared to the FL-CHESDA-WSN, DA-WSN-MLOA, and IDAF-ASLPPRR benchmark methods. The MOCHSAPGAN-AVO method showed a 37.94%, 22.96%, and 18.35% improved packet delivery ratio compared to the FL-CHESDA-WSN, DA-WSN-MLOA, and IDAF-ASLPPRR benchmark methods.

Cherappa et al. [31] proposed an expedient routing protocol-based energy-efficient clustering technique using an adaptive sailfish optimization algorithm (ASFO). The ASFO used K-ASFO to select optimal CH from suitable nodes. The proposed ASFO approach transferred data efficiently using the CMRP routing protocol. The performance of ASFO was simulated in the MATLAB tool. The simulation result of ASFO was compared with KSOA and WOA. The performance of ASFO was evaluated in terms of PDR, packet delay, throughput, power consumption, network lifetime, and PLR. The overall accuracy of the proposed ASFO technique was 93.19%, which was better than the KSOA and WOA benchmark works.

Many existing methods for CH selection in WSN have been developed, utilizing various optimization algorithms. Existing approaches’ limitations include increasing energy consumption, selecting inappropriate CH in the network, and communication overhead. The significant limitations of existing works are listed in Table 1.

## 3. System Model

### 3.1. Network Model

**Figure 1:**
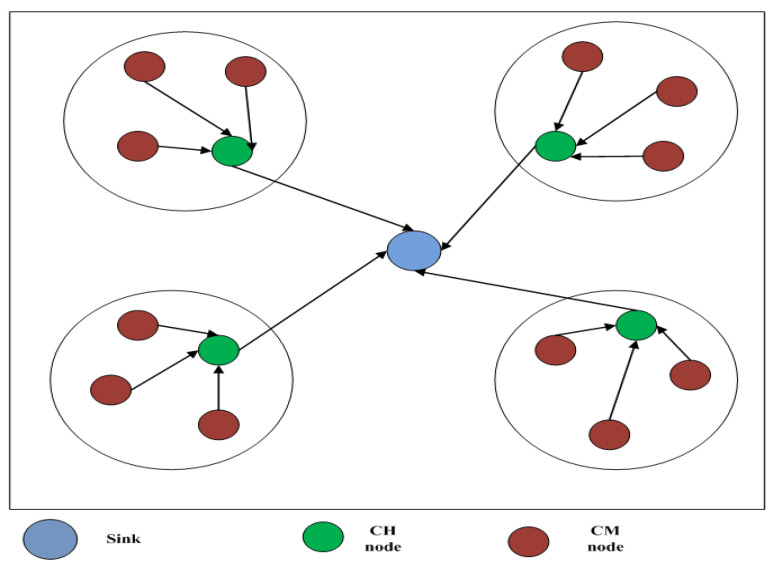
SWARAM network model.

In SWARAM, the network is made of several nodes that are scattered randomly in network space. Every node in the network can be a cluster head (CH) or cluster member (CM). The CM sends data to CH by sensing around, and CH sends receives data to the base station (BS) or sink node by aggregating them. The BS performs analysis and makes appropriate decisions over CH data. The proposed SWARAM optimization algorithm performs CH selection after executing in the sink node. Later, the cluster is created with nearby nodes of CH. The network model is presented in Figure 1.

The assumptions of the SWARAM network model are presented below:

- The nodes in the network are scattered randomly.
- Every node has a unique identifier to differentiate it from other nodes.
- All nodes are homogeneous in terms of energy and computational power.
- The sink node is in the center of the network.
- All nodes are aware of the sink node location in a network.
- The sink node receives aggregated data packets from the CH obtained from the cluster’s CMs.

### 3.2. Energy Model

**Figure 2:**
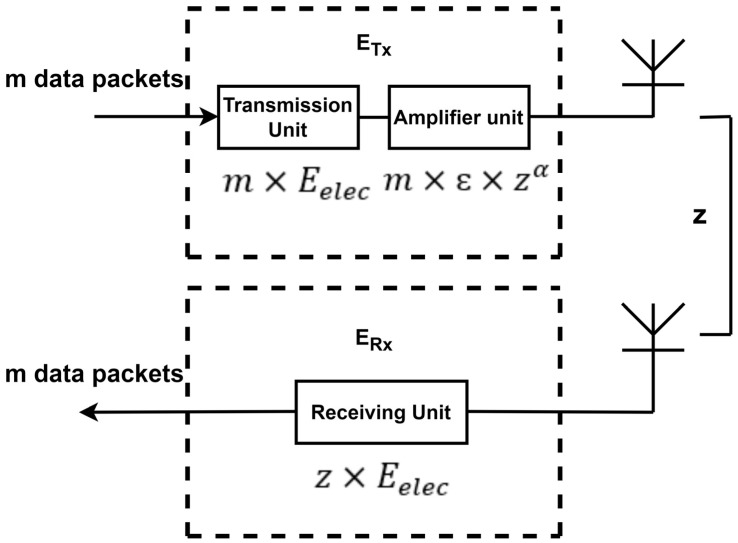
SWARAM energy model.

Energy consumption becomes challenging for sending and receiving data in network space. It is controlled by considering both free-space and multi-path models. The proposed SWARAM protocol adapts a simplified energy model for transmitting data from the participant to the sink node [32]. The SWARAM energy model is presented in Figure 2. In SWARAM, two nodes, ‘p’ and ‘q’, transmit data of ‘m’ bits with respect to distance z; (x, y) is computed in Equation (1).

(1) $$E_{TX}\left(m,z\right)=mE_{elec}+m\varepsilon z{\left(p,q\right)}^{\alpha \;}\;=\{\begin{array}{c} mE_{elec}+m\varepsilon_{\mathrm{ft}}z{\left(p,q\right)}^{2\;}\;\;\;where\;z\left(p,q\right)<z_{0}\\ mE_{elec}+m\varepsilon_{\mathrm{mp}}z{\left(p,q\right)}^{4}\;\;where\;z\left(p,q\right)\geq \;z_{0} \end{array}\;$$

The distance between ‘p’ and ‘q’ nodes is ‘z’, which is shorter than $z_{0}$ ; then, it uses a model of free space to compute energy consumption. Otherwise, it uses a multipath model. The boundary value of distance is calculated in Equation (2).

(2) $$z0=\sqrt{\frac{\varepsilon_{fm}}{\varepsilon_{mp}}}$$

Moreover, the energy that node ‘p’ consumes to transfer ‘m’ bits of data to node ‘q’ is calculated using Equation (3).

(3) $$E_{RX}\left(z\right)=zE_{elec}$$

## 4. The Proposed SWARAM Protocol

This paper proposes an osprey optimization algorithm based on efficient cluster head selection (SWARAM) in wireless sensor networks to address network lifetime and end-to-end delay issues. It has two phases; in the first phase, CH selection is achieved using the osprey optimization algorithm (OOA), and the cluster is formed with a group of nodes in the second phase using Euclidean distance. Therefore, it improves the overall performance of the network. Table 2 presents the nomenclature of SWARAM.

### 4.1. Osprey Optimization Algorithm Based CH Selection

Osprey optimization algorithm (OOA) is a bio-inspired metaheuristic algorithm, which is also termed water hawk [33]. It is simulated based on the behavior of ospreys to solve complex problems. The weight, length, and wingspan of ospreys are 0.9–2.1 kg, 50–66 cm, and 127–180 cm. Ospreys mainly eat fish by hunting prey, which weighs up to 2 kg. Ospreys can detect fish underwater by flying 40 m above the water’s surface. Then, they catch fish with their feet by diving into the water and carrying it to a safe place to eat. The hunting and eating behavior of natural ospreys are employed in designing the optimization algorithm. The intelligent behavior of ospreys as a mathematical model is used for developing the OOA technique.

#### 4.1.1. Initialization

The OOA is a population-based approach that provides the best solution in a problem-solving space through a number of iterations [33]. The osprey is treated as a sensor node in the osprey population and is represented using a matrix in Equation (4). Each osprey represents a sample of the population, contributing information about its location to the solution of the problem.

(4) $$O={\left[\begin{array}{c} \begin{array}{c} O_{1}\\ \vdots \end{array}\\ \begin{array}{c} O_{i}\\ \vdots \end{array}\\ O_{n} \end{array}\right]}_{n\times m}={\left[\begin{array}{ccccc} o_{1,1} & \cdots & o_{1,j} & \cdots & o_{1,m}\\ \vdots & \ddots & \vdots & \ddots & \vdots \\ o_{i,1} & \ldots & o_{i,j} & \ldots & o_{i,m}\\ \vdots & \ddots & \vdots & \ddots & \vdots \\ o_{n,1} & \ldots & o_{n,j} & \ldots & o_{n,m} \end{array}\right]}_{n\times m}$$

The network contains ‘n’ sensor nodes that are initialized randomly using Equation (5).

(5) $$O_{i,j}={LB}_{j}+r_{i,j}\cdot \left({upb}_{j}-{lob}_{j}\right)\;\;\;\;\;i=1,2,\ldots ,n,\;\;\;\;j=1,2,\ldots ,m,$$

where ‘O’ represents the osprey population matrix, $O_{i}$ and $O_{i,j}$ are ith osprey and ith osprey at the jth dimension, n and m are the number of ospreys and the number of problem variables, $r_{i,j}$ represents a random value between 0 and 1, $upb_{j}$ and $lob_{j}$ are the upper bound and lower bound of the jth problem variable.

The objective function generates set of values (osprey) by evaluating the problem. The estimated values of the problem are presented using a vector in Equation (6).

(6) $$F={\left[\begin{array}{c} F_{1}\\ \vdots \\ F_{i}\\ \vdots \\ F_{n} \end{array}\right]}_{n\times 1}={\left[\begin{array}{c} F\left(O_{1}\right)\\ \vdots \\ F\left(O_{i}\right)\\ \vdots \\ F\left(O_{n}\right) \end{array}\right]}_{n\times 1}$$

where F indicates a set of values of objective function and $F_{i}$ represents ith osprey value of objective function. The objective function of estimated values is used to evaluate the quality of the candidate solution. In the iteration procedure, the best and worst values are considered to select the best and word members. For each iteration, the osprey position is evaluated and updated.

#### 4.1.2. Exploration Phase

Ospreys can hunt fish underwater by identifying their position in water due to their sharp eyesight. Once the place of fish is identified, osprey attack and hunt fish by diving into the water. In the initial stage of OOA, the population is updated using a model inspired by the actions of ospreys. The position of the osprey is updated in search space due to modeling of the osprey attack on fish, which strengthens the OOA exploration power for identifying and escaping from local optima.

The position of other ospreys in the search space considers the higher objective value of underwater fish for each osprey. The group of fish for each osprey is formulated in Equation (7).

(7) $$Fpos_{i}=\left\{O_{w}|w\in \left\{1,2,\ldots ,n\right\}\wedge FH_{w}<FH_{i}\right\}\cup \left\{O_{best}\right\},$$

where $Fpos_{i}$ represents the group of fish for ith osprey and the best osprey represented by $O_{best}$ .

The osprey attacks underwater fish by finding its position randomly. Using Equation (8), the new location of the osprey in the search space is determined by the osprey’s movement towards the fish. If the new value of the osprey position improves the previous objective value, then replace the osprey’s last position using Equation (9).

(8a) $$o_{i,j}^{pos1}=0_{i,j}+ran_{i,j}\cdot \left(SF_{i,j}-R_{i,j}\cdot y_{i,j}\right),$$

(8b) $$o_{i,j}^{pos1}=\{\begin{array}{c} o_{i,j}^{pos1},lob_{j}\leq o_{i,j}^{pos1}\leq upb_{j};\\ lob_{j},o_{i,j}^{pos1}<lob_{j};\\ upb_{j},o_{i,j}^{pos1}>upb_{j}; \end{array}$$

(9) $$O_{i}=\{\begin{array}{c} O_{i}^{pos1},FH_{i}^{pos1}<FH_{i};\\ O_{i},else, \end{array}$$

where $O_{i}^{pos1}$ indicates the new position of ith osprey as per OOA first phase, $O_{i,j}^{pos1}$ represents the jth dimension, $FH_{i}^{pos1}$ represents objective function value, $SF_{i}$ indicates selected fish for ith osprey, $SF_{i,j}$ is jth dimension, the random interval [0,1] is defined by $ran_{i,j}$ , and the random number between 1 and 2 is indicated by $R_{i,j}$ .

#### 4.1.3. Exploitation Phase

The osprey finds a safe place to eat fish after hunting. The population update in the second phase of OOA is modeled based on the natural behavior of ospreys. The position of the osprey in the search space alters due to carrying fish to a safe place to eat, which strengthens the OOA’s exploitation capacity for local search and better solutions.

The osprey’s behavior is simulated to calculate the random position for each osprey in the population to eat hunted fish in the OOA design phase using Equation (10). The osprey’s prior position is updated based on a better value of the objective function derived in Equation (11).

(10a) $$o_{i,j}^{pos2}=o_{i,j}+\frac{lob_{j}+ran_{i,j}\cdot \left(upb_{j}-low_{j}\right)}{k},\;i=1,\;2,\;3,\ldots ,n,\;j=1,2,\ldots ,m,\;k=1,\;2,\ldots ,T,$$

(10b) $$x_{i,j}^{pos2}=\{\begin{array}{c} o_{i,j}^{pos2},\;lob_{j}\leq o_{i,j}^{pos2}\leq upb_{j};\\ lob_{j},\;o_{i,j}^{pos2}<lob_{j};\\ upb_{j},\;o_{i,j}^{pos2}>upb_{j}; \end{array}$$

(11) $$O_{i}=\{\begin{array}{c} O_{i}^{pos2},\;FH_{i}^{pos2}<FH_{i};\\ O_{i},\;else, \end{array}$$

where $O_{i}^{pos2}$ indicates the new position of ith osprey as per OOA second phase, $O_{i,j}^{pos2}$ represents the jth dimension, $FH_{i}^{pos2}$ represents objective function value, the random interval [0,1] defined by $ran_{i,j}$ , k denotes the algorithm iteration counter, and K indicates the count of iterations.

#### 4.1.4. Fitness Function

The fitness function is a crucial component used to obtain the location of fish to hunt by osprey. In SWARAM, we consider parameters such as Residual Energy (RER) and distance between node and Base station (BS) or Sink node (Distance) to find a potential solution.

- **Residual Energy (RER)**

The RER specifies the energy left in network nodes after it has been in operation [34]. The RER is computed from the initial power and remaining power of the node. The RER is calculated using Equation (12).

(12) $$RER\left(O_{i}\right)=\frac{Energy_{avail}}{Energy_{intial}}$$

where $Energy_{avail}$ represents available energy and $Energy_{intial}$ indicates initial energy.

- **Distance**

The distance parameter selects the optimal node to become CH based on the proximity between sensor node $O_{i}$ and sink node and is obtained using Euclidean distance [35]. The distance is calculated using Equation (13).

(13) $$dis\left(O_{i},sink\right)=\sqrt{\sum_{i=1}^{n}{\left(sink-O_{i}\right)}^{2}}$$

The osprey current position of fitness function is obtained using Equation (14).

(14) $${O_{i}}_{\left(fitness\right)}=0.5\times (1-RER\left(O_{i}\right)+0.5\times \left(1-dis\left(O_{i}\right)\right)$$

The osprey position is computed in each iteration and checks with the previous value of the objective function. If the osprey reaches the best position in the current iteration compared to the last iteration, then that node is considered CH in the corresponding iteration. Algorithm 1 provides pseudocode for the CH selection procedure.
**Algorithm 1:** SWARAM-based CH selection algorithmInput: Network population size set to ‘n’ nodes and total count of iterations ‘T’ Output: optimal position of osprey acts as CH node. 1: Initialize network population randomly using Equations (1) and (2). 2: The objective function is computed using Equation (3). 3: For t = 1 to T do. 4: For i = 1 to n do. //exploration phase 5: The fish position is updated for member of OOA using Equation (4). 6: The 
SF
 is determined randomly using ith osprey. 7: Osprey’s new position is computed using Equation (8a). 8: The boundary condition is verified using Equation (8b). 9: ith osprey position is updated using Equation (6). //exploitation phase 10: the new position of osprey is computed using Equation (10a). 11: The boundary condition is verified for new position of osprey using Equation (10b). 12: Update the position of osprey using Equation (8). 13: Evaluate the fitness function using Equation (11). 14: If osprey reaches optimal position in network, then 15:  Best candidate osprey act as CH 16: else 17:  Go to step 1. 18: END for 19: END for 20: Return candidate CH.

### 4.2. Cluster Formation

The sensor nodes ‘n’ are deployed in the network and grouped as clusters after the CH selection. The CH selection is crucial to address energy efficiency and network lifetime. The clustering of nodes is achieved using Euclidean distance. In clustering, Euclidean distance is a fundamental component of cluster formation for effectiveness in the network. Each node evaluates the distance from candid CH in the network area. All nodes together select CH intelligently by considering proximity. It is suitable for scenarios where energy consumption and node communication are optimized. It is given in Equation (15).

(15) $$\mathrm{dis}\left(O_{i},O_{j}\right)=\sqrt{\sum_{i=1}^{n}{\left(O_{j}-O_{i}\right)}^{2},}$$

where $O_{i}$ and $O_{j}$ are two different nodes in network area.

**Figure 3:**
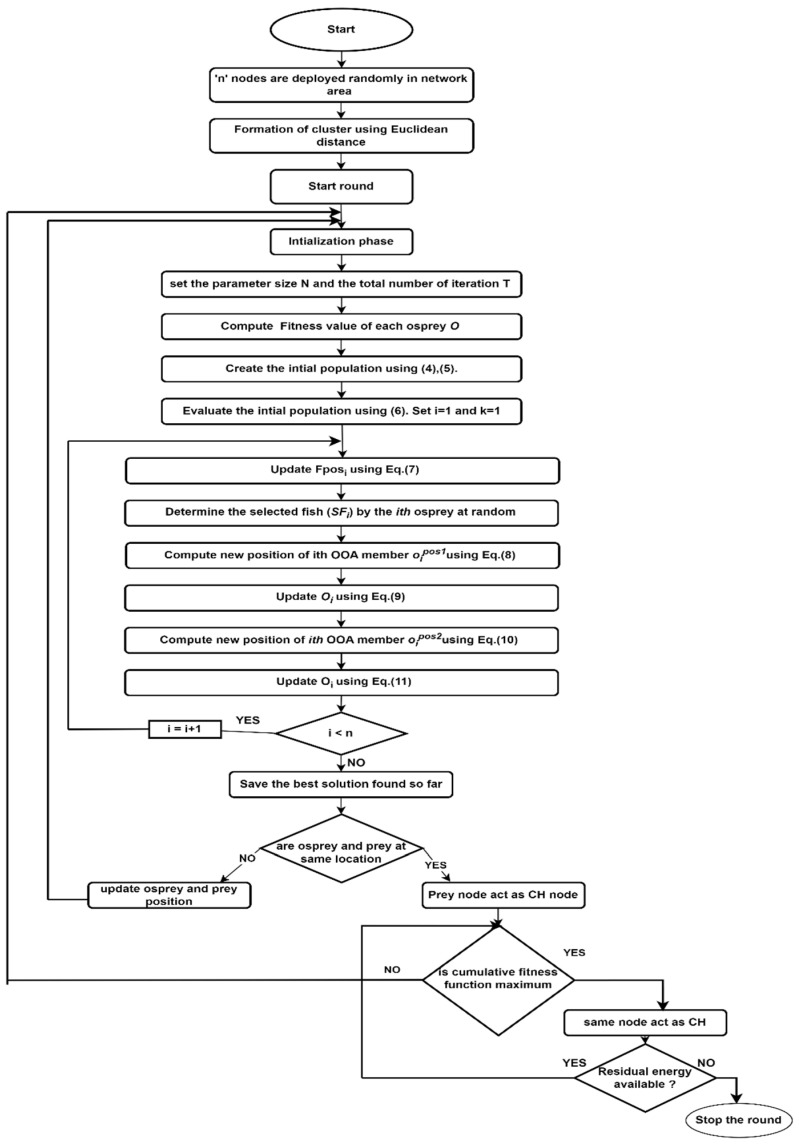
SWARAM workflow.

The flow of CH selection is represented in Figure 3.

## 5. Simulation Results

The performance of the proposed SWARAM approach is obtained using MATLAB 2019a over a 500 m × 500 m network simulation environment [36,37,38]. Nodes are distributed using a flat network model in the network region. The efficiency of SWARAM techniques is matched with benchmark techniques EECHS-ARO, HSWO, and EECHIGWO using various parameters. All benchmark techniques use the same set of input parameters for simulation. The parameters considered for comparison are energy consumption, count of alive nodes, communication overhead, and number of packets received by the sink node. The simulation square space 500 m × 500 m is considered with 400 nodes disseminated arbitrarily in the network. The sink node is placed at 250 m 250 m center of the network and ensures every sensor node maintains at least one neighbor node. Every node in the network is able to send data packets to BS using a single hop. All nodes have the same communication range and exhibit heterogeneity. All nodes are initialized with 1 Joule battery energy. A total of 3000 simulation rounds are considered in the network. The network parameters with values are given in Table 3.

### 5.1. Network Lifetime

**Figure 4:**
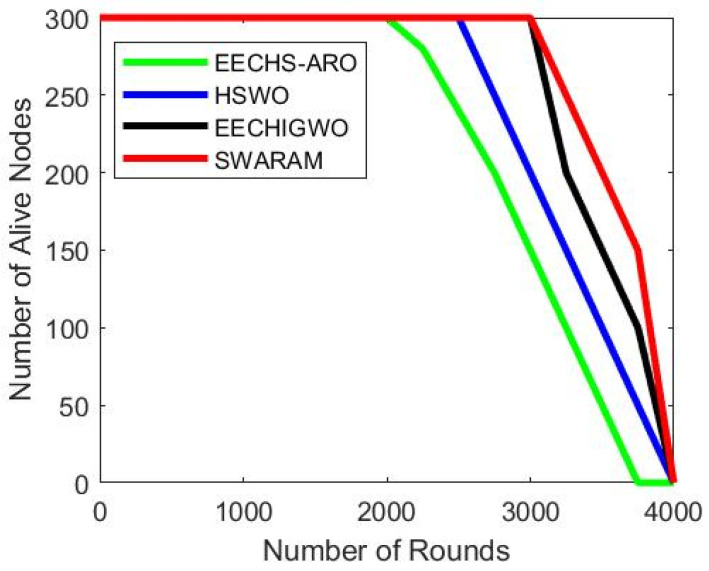
Network lifespan.

The alive nodes determine network sustainability on the network. Figure 4 demonstrates the number of active nodes concerning several network rounds. The analysis of alive nodes is observed in the matter of network rounds. The performance of the proposed SWARAM approach is examined and compared with EECHS-ARO, HSWO, and EECHIGWO benchmark techniques in terms of alive nodes. It is also observed that the alive nodes of EECHS-ARO, HSWO EECHIGWO, and SWARAM are 150, 200, 300, and 300 for the network around 3000. Additionally, it is observed that during network round 4000, the count of alive nodes has reached zero. As the number of network rounds increases, there is a discernible drop in the number of alive nodes. However, the active nodes of the proposed SWARAM approach outperformed compared to EECHS-ARO, HSWO, and EECHIGWO techniques.

The number of alive nodes of different techniques associated with several network rounds is listed in Table 4. As the number of network rounds increases, there is a discernible drop in the number of alive nodes.

### 5.2. Average Energy Consumption

**Figure 5:**
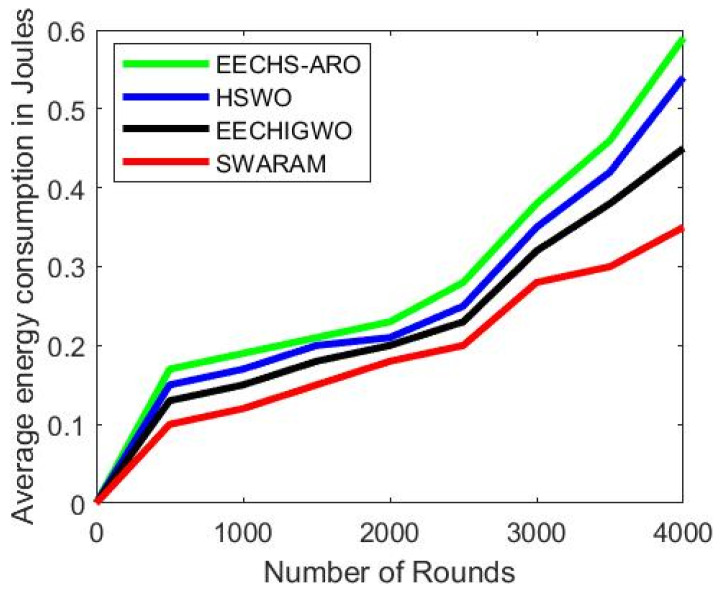
Average energy consumption.

The sensor nodes consume energy for transmitting data to destination nodes. Figure 5 illustrates the overall energy consumption of nodes concerning network rounds. The energy consumption of the proposed SWARAM approach is examined and compared with existing EECHS-ARO, HSWO, and EECHIGWO approaches. It is noticed that the energy consumption of EECHS-ARO, HSWO, EECHIGWO, and SWARAM are 0.59 J, 0.54 J, 0.45 J, and 0.35 J, respectively. The proposed SWARAM protocol reduces energy consumption by 24%, 20%, and 10% compared to EECHS-ARO, HSWO, and EECHIGWO methods for 4000 network rounds. The SWARAM technique takes less convergence time for the CH selection process. It is due to SWARAM considering RER and Distance parameters to compute fitness function. It is evident that when a network’s round count rises, so does the energy usage.

Table 5 shows the average energy usage of many optimization strategies in relation to network rounds. The proposed approach, SWARAM, optimizes energy consumption better than existing approaches. The energy consumption of nodes increases when the network iteration process rises.

### 5.3. Average Communication Overhead

**Figure 6:**
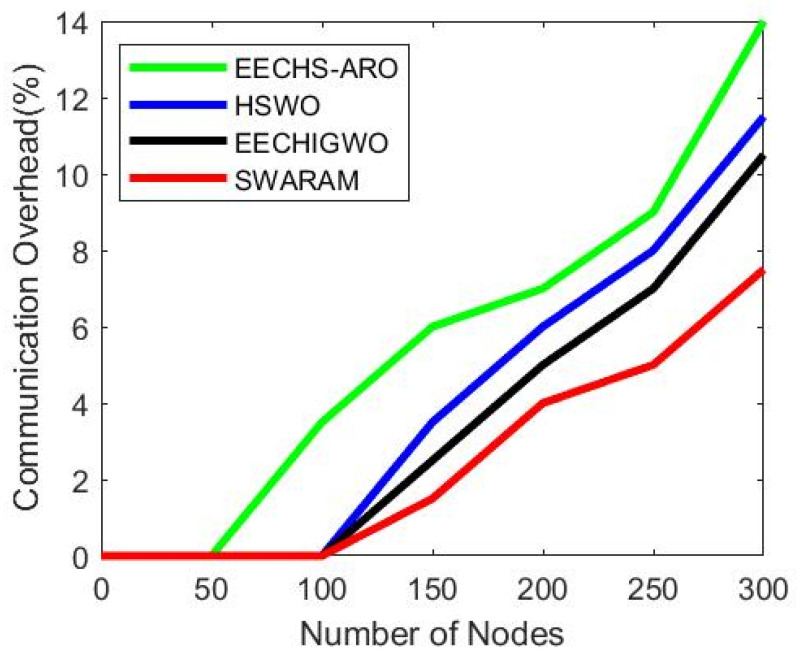
Performance analysis of average communication overhead varying number of nodes.

The proposed SWARAM approach communication overhead is analyzed over existing EECHS-ARO, HSWO, and EECHIGWO approaches. The communication overhead is measured by varying counts of nodes on a fixed network area. Figure 6 illustrates the analysis of communication overhead for several network nodes. The average communication overheads of EECHS-ARO, HSWO, EECHIGWO, and SWARAM are 14%, 11.5%, 10.5, and 7.5 considering 300 nodes in the network. The overall average communication overhead is reduced in SWARAM protocol by 6.5%, 4%, and 3%, respectively, over EECHS-ARO, HSWO, and EECHIGWO techniques. Compared with existing optimization techniques, the proposed SWARAM protocol decreases communication overhead caused by picking a CH node in the clusters and quickly rotates the CH node. It is noticeable that communication overhead for EECHS-ARO, HSWO, EECHIGWO, and SWARAM methods increases substantially as the node size increases.

Table 6 presents the average communication overhead of various techniques associated with a number of nodes. The communication overhead increases significantly as the number of nodes on a fixed network region rises.

### 5.4. Analysis of Average Packet Delivery to Sink

**Figure 7:**
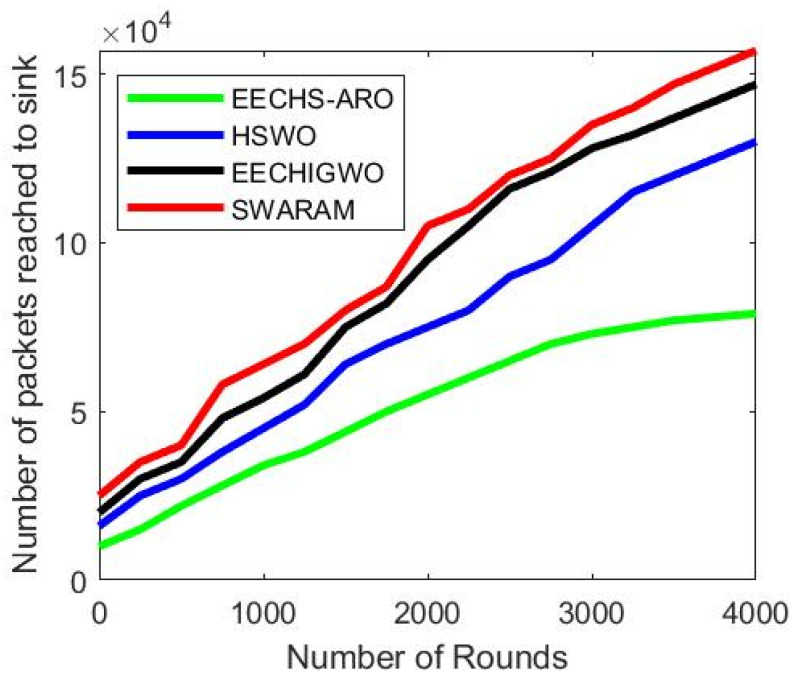
Performance analysis of average packet delivery to sink with varied network rounds.

The proposed SWARAM approach performance regarding average packet delivery to sink node is analyzed over existing EECHS-ARO, HSWO, and EECHIGWO approaches. Increasing network rounds carry out the analysis of average packet delivery on a fixed network area. Figure 7 illustrates the calculation of average packet delivery to sink for several network rounds. The average packet deliveries of EECHS-ARO, HSWO, EECHIGWO, and SWARAM are 79,000, 130,000, 147,000, and 157,000 for the 4000 network rounds. The overall average packet delivery to the sink node is improved in SWARAM protocol by 78%, 27%, and 10%, respectively, over EECHS-ARO, HSWO, and EECHIGWO techniques. The proposed SWARAM protocol improves average packet delivery to sink due to selecting a CH node in the clusters. It reduces data packet loss during transmission over the network compared to other optimization algorithms. It is noticeable that the average packet delivery to sink of SWARAM is effective regardless of network density compared with EECHS-ARO, HSWO, and EECHIGWO.

Table 7 presents the average packet delivery of various techniques associated with different network rounds. It is noticeable that packet delivery to sink increases substantially as the network round increases in the network area.

## 6. Conclusions

The limited battery life of sensors becomes the main challenge in WSN. Energy conservation is a significant task to enhance the performance of the network. Transmitting data among nodes using clustering protocol is a good approach. Many existing strategies have been proposed for CH selection utilizing an optimization algorithm. However, converge time is extended during CH selection using various optimization algorithms. To solve this issue, this paper proposes an osprey optimization algorithm (SWARAM) to choose optimal CH in the network. The proposed SWARAM approach considers distance and RER parameters to compute the fitness function. The proposed SWARAM approach consists of two phases, namely, cluster formation and CH selection. The nodes are clustered using Euclidean distance before the CH node is selected using the SWARAM technique. Simulation of the proposed SWARAM algorithm is carried out in the MATLAB2019a tool. The performance of the SWARAM algorithm is compared with existing EECHS-ARO, HSWO, and EECHIGWO CH selection algorithms. The suggested SWARAM improves the packet delivery ratio and network lifetime by 10% and 10%, respectively. Consequently, the overall performance of the network is improved.

In future work, the performance of the SWARAM scheme can be tested in a real-time environment by considering various other performance parameters such as mobility, security, fault tolerance, and load balancing. In addition, we can develop hybrid algorithms to choose optimal CH.

## Figures and Tables

**Table 1:** Existing optimization-based CH selection approaches.

S.No	Authors	Proposed Algorithms	Advantages	Limitations
1	Chaurasia et al. [22]	Dragonfly Algorithm (DA)	Enhanced energy efficiency by 8–10%.	Network clustering takes longer.
2	Rami Reddy et al. [23]	Improved Grey Wolf Optimization (IGWO)	Improved network stability by 16–19%.	Energy consumption increases during the process of CH selection.
3	Samiayya et al. [24]	Hybrid Snake Whale Optimization (HSWO)	Minimized normalized energy consumption by 5–10%.	Early energy depletion occurs in sensor nodes during the CH selection process.
4	Ramalingam et al. [25]	Artificial Rabbits Optimization Algorithm (AROA)	Extended network lifetime and throughput by 15% and 5%.	Network clustering takes longer.
5	Arunachalam et al. [26]	Squirrel Search Optimization (SSO)	Extended network lifetime and throughput and minimized energy consumption by 17.92%, 13.48% and 15.29%.	Takes more time to converge.
6	Abraham et al. [27]	Flamingo Search Algorithm (FSA)	Enhanced network lifetime and energy efficiency.	All CH nodes being selected by algorithm is not optimal.
7	Ambareesh et al. [28]	Hybrid Jarratt Butterfly Optimization (HJBO)	Improved throughput and packet delivery ratio by 5% and 6%.	It takes longer to choose CH item.
8	Jaya Pratha et al. [29]	Hybrid Mutualism Mechanism-inspired Butterfly and Flower Pollination Optimization Algorithm (HMMB-FPOA)	Improved network lifetime and packet delivery ratio by 16.21% and 13.28%.	HMMB-FPOA approach is suitable for specific scenarios.
9	Sindhuja et al. [30]	African vulture optimization (MOCHSAPGAN-AVO)	The packet delivery ratio and throughput are improved.	Energy consumption increases during the clustering process.
10	Cherappa et al. [31]	Adaptive Sailfish Optimization (ASFO)	Improved overall network performance 93.19%.	Takes more time to converge.

**Table 2:** Nomenclature of SWARAM algorithm.

Notation	Definition
RER	Residual energy
*O*	Osprey
$r_{i,j}$	Random number in the interval [0,1]
$O_{i}^{pos1}$	Current position of *i*th osprey in phase 1
$SF_{i}$	Selected fish for *i*th osprey
upb	Upper bound
low	Lower bound
$O_{best}$	Best candidate solution
$R_{i,j}$	Random number form the set [1,2]
$O_{i}^{pos2}$	Current position of *i*th osprey in phase 2
*T*	Total number of iterations
*k*	Algorithm iteration counter
*BS*	Base station
*CH*	Cluster head
*CM*	Cluster member

**Table 3:** Simulation Parameters.

Parameters	Values
Simulation tool	MATLAB R2019a
Network size	500 m × 500 m
Sink position	(250 m, 250 m)
Maximum Iterations	3000
Packet size	4000 bits
Node initial energy	1.2 J
$\varepsilon_{ec}$	50 nJ/bit
$\varepsilon_{friss}$	$10\;\mathrm{pJ}/\mathrm{bit}/m^{2}$
Node count	400
$\varepsilon_{mpm}$	$0.0013\;\mathrm{pJ}/\mathrm{bit}/m^{4}$
$E_{\mathrm{elec}}$	50 nj/bit
$E_{receive}$	0.055 μj/bit
$E_{\mathrm{aggregate}}$	0.00012 μj/bit
$E_{\mathrm{amp}}$	$10\;\;\mathrm{pj}/\mathrm{bit}/m^{2}$
$E_{\mathrm{transmit}}$	0.039 μj/bit

**Table 4:** Number of alive nodes vs. number of network rounds.

Number of Rounds	EECHS-ARO	HSWO	EECHIGWO	SWARAM
0	300	300	300	300
250	300	300	300	300
500	300	300	300	300
750	300	300	300	300
1000	300	300	300	300
1250	300	300	300	300
1500	300	300	300	300
1750	300	300	300	300
2000	300	300	300	300
2250	280	300	300	300
2500	240	300	300	300
2750	200	250	300	300
3000	150	200	300	300
3250	100	150	200	250
3500	50	100	150	200
3750	0	50	100	150
4000	0	0	0	0

**Table 5:** Average energy consumption.

Number of Rounds	EECHS-ARO	HSWO	EECHIGWO	SWARAM
0	0	0	0	0
500	0.17	0.15	0.13	0.10
1000	0.19	0.17	0.15	0.12
1500	0.21	0.20	0.18	0.15
2000	0.23	0.21	0.20	0.18
2500	0.28	0.25	0.23	0.20
3000	0.38	0.35	0.32	0.28
3500	0.46	0.42	0.38	0.30
4000	0.59	0.54	0.45	0.35

**Table 6:** Communication Overhead.

Number of Nodes	EECHS-ARO (%)	HSWO (%)	EECHIGWO (%)	SWARAM (%)
0	0	0	0	0
50	0	0	0	0
100	3.5	0	0	0
150	6	3.5	2.5	1.5
200	7	6	5	4
250	9	8	7	5
300	14	11.5	10.5	7.5

**Table 7:** Packet delivery to sink.

Number of Rounds	EECHS-ARO	HSWO	EECHIGWO	SWARAM
0	10,000	16,000	20,000	25,000
250	15,000	25,000	30,000	35,000
500	22,000	30,000	35,000	40,000
750	28,000	38,000	48,000	58,000
1000	34,000	45,000	54,000	64,000
1250	38,000	52,000	61,000	70,000
1500	44,000	64,000	75,000	80,000
1750	50,000	70,000	82,000	87,000
2000	55,000	75,000	95,000	105,000
2250	60,000	80,000	105,000	110,000
2500	65,000	90,000	116,000	120,000
2750	70,000	95,000	121,000	125,000
3000	73,000	105,000	128,000	135,000
3250	75,000	115,000	132,000	140,000
3500	77,000	120,000	137,000	147,000
3750	78,000	125,000	142,000	152,000
4000	79,000	130,000	147,000	157,000

## Data Availability

The dataset used for this work was randomly generated in MATLAB.

## References

[1] Heinzelman W.R., Chandrakasan A., Balakrishnan H. (2000). Energy-efficient communication protocol for wireless microsensor networks. Proceedings of the 33rd Annual Hawaii International Conference on System Sciences.

[2] Nauman A., Qadri Y.A., Amjad M., Zikria Y.B., Afzal M.K., Kim S.W. (2020). Multimedia Internet of Things: A comprehensive survey. IEEE Access.

[3] Sennan S., Ramasubbareddy S., Balasubramaniyam S., Nayyar A., Abouhawwash M., Hikal N.A. (2021). T2FL-PSO: Type-2 fuzzy logic-based particle swarm optimization algorithm used to maximize the lifetime of Internet of Things. IEEE Access.

[4] Kassab W.A., Darabkh K.A. (2020). A–Z survey of Internet of Things: Architectures, protocols, applications, recent advances, future directions and recommendations. J. Netw. Comput. Appl..

[5] Thuluva A.S.S., Somanathan M.S., Somula R., Sennan S., Burgos D. (2021). Secure and efficient transmission of data based on Caesar Cipher Algorithm for Sybil attack in IoT. EURASIP J. Adv. Signal Process..

[6] Roy S., Mazumdar N., Pamula R. (2021). An optimal mobile sink sojourn location discovery approach for the energy-constrained and delay-sensitive wireless sensor network. J. Ambient. Intell. Humaniz. Comput..

[7] Palanisamy S., Sankar S., Somula R., Deverajan G.G. (2021). Communication trust and energy-aware routing protocol for WSN using DS theory. Int. J. Grid High Perform. Comput. (IJGHPC).

[8] Kandris D., Nakas C., Vomvas D., Koulouras G. (2020). Applications of wireless sensor networks: An up-to-date survey. Appl. Syst. Innov..

[9] Shahraki A., Taherkordi A., Haugen Ø., Eliassen F. (2020). Clustering objectives in wireless sensor networks: A survey and research direction analysis. Comput. Netw..

[10] Somula R., Cho Y., Mohanta B.K. (2023). EACH-COA: An Energy-Aware Cluster Head Selection for the Internet of Things Using the Coati Optimization Algorithm. Information.

[11] Elsmany EF A., Omar M.A., Wan T.C., Altahir A.A. (2019). EESRA: Energy efficient scalable routing algorithm for wireless sensor networks. IEEE Access.

[12] Sankar S., Ramasubbareddy S., Luhach A.K., Alnumay W.S., Chatterjee P. (2022). NCCLA: New caledonian crow learning algorithm based cluster head selection for Internet of Things in smart cities. J. Ambient. Intell. Humaniz. Comput..

[13] Iwendi C., Maddikunta PK R., Gadekallu T.R., Lakshmanna K., Bashir A.K., Piran M.J. (2021). A metaheuristic optimization approach for energy efficiency in the IoT networks. Softw. Pract. Exp..

[14] Hellaoui H., Koudil M., Bouabdallah A. (2020). Energy efficiency in security of 5G-based IoT: An end-to-end adaptive approach. IEEE Internet Things J..

[15] Metallidou C.K., Psannis K.E., Egyptiadou E.A. (2020). Energy efficiency in smart buildings: IoT approaches. IEEE Access.

[16] Kennedy J., Eberhart R. (1995). Particle swarm optimization. Proceedings of the ICNN’95-International Conference on Neural Networks.

[17] Karaboga D., Basturk B. (2007). Artificial bee colony (ABC) optimization algorithm for solving constrained optimization problems. International Fuzzy Systems Association World Congress.

[18] Chopra N., Ansari M.M. (2022). Golden jackal optimization: A novel nature-inspired optimizer for engineering applications. Expert Syst. Appl..

[19] Dehghani M., Montazeri Z., Trojovská E., Trojovský P. (2023). Coati Optimization Algorithm: A new bio-inspired metaheuristic algorithm for solving optimization problems. Knowl.-Based Syst..

[20] Faramarzi A., Heidarinejad M., Mirjalili S., Gandomi A.H. (2020). Marine Predators Algorithm: A nature-inspired metaheuristic. Expert Syst. Appl..

[21] Mirjalili S., Lewis A. (2016). The whale optimization algorithm. Adv. Eng. Softw..

[22] Chaurasia S., Kumar K., Kumar N. (2023). Mocraw: A meta-heuristic optimized cluster head selection based routing algorithm for wsns. Ad Hoc Netw..

[23] Rami Reddy M., Ravi Chandra M.L., Venkatramana P., Dilli R. (2023). Energy-efficient cluster head selection in wireless sensor networks using an improved grey wolf optimization algorithm. Computers.

[24] Samiayya D., Radhika S., Chandrasekar A. (2023). An optimal model for enhancing network lifetime and cluster head selection using hybrid snake whale optimization. Peer-to-Peer Netw. Appl..

[25] Ramalingam R., Saleena B., Basheer S., Balasubramanian P., Rashid M., Jayaraman G. (2023). EECHS-ARO: Energy-efficient cluster head selection mechanism for livestock industry using artificial rabbits optimization and wireless sensor networks. Electron. Res. Arch..

[26] Arunachalam N., Shanmugasundaram G., Arvind R. (2021). Squirrel search optimization-based cluster head selection technique for prolonging lifetime in WSN’s. Wirel. Pers. Commun..

[27] Abraham R., Vadivel M. (2023). An Energy Efficient Wireless Sensor Network with Flamingo Search Algorithm Based Cluster Head Selection. Wirel. Pers. Commun..

[28] Ambareesh S., Kantharaju H.C., Sakthivel M. (2023). A novel Fuzzy TOPSIS based hybrid jarratt butterfly optimization for optimal routing and cluster head selection in WSN. Peer-to-Peer Netw. Appl..

[29] Pratha S.J., Asanambigai V., Mugunthan S.R. (2023). Hybrid Mutualism Mechanism-Inspired Butterfly and Flower Pollination Optimization Algorithm for Lifetime Improving Energy-Efficient Cluster Head Selection in WSNs. Wirel. Pers. Commun..

[30] Sindhuja M., Vidhya S., Jayasri B.S., Shajin F.H. (2023). Multi-objective cluster head using self-attention based progressive generative adversarial network for secured data aggregation. Ad Hoc Netw..

[31] Cherappa V., Thangarajan T., Meenakshi Sundaram S.S., Hajjej F., Munusamy A.K., Shanmugam R. (2023). Energy-Efficient Clustering and Routing Using ASFO and a Cross-Layer-Based Expedient Routing Protocol for Wireless Sensor Networks. Sensors.

[32] Abu Salem A.O., Shudifat N. (2019). Enhanced LEACH protocol for increasing a lifetime of WSNs. Pers. Ubiquitous Comput..

[33] Trojovský P., Dehghani M. (2023). Osprey Optimization Algorithm: A new bio-inspired metaheuristic algorithm for solving engineering optimization problems. Front. Mech. Eng..

[34] Sankar S., Srinivasan P., Luhach A.K., Somula R., Chilamkurti N. (2020). Energy-aware grid-based data aggregation scheme in routing protocol for agricultural internet of things. Sustain. Comput. Inform. Syst..

[35] Sahoo B.M., Amgoth T., Pandey H.M. (2020). Particle swarm optimization based energy efficient clustering and sink mobility in heterogeneous wireless sensor network. Ad Hoc Netw..

[36] Rawat P., Chauhan S. (2021). Probability based cluster routing protocol for wireless sensor network. J. Ambient. Intell. Humaniz. Comput..

[37] Bhola J., Soni S., Cheema G.K. (2020). Genetic algorithm based optimized leach protocol for energy efficient wireless sensor networks. J. Ambient. Intell. Humaniz. Comput..

[38] Rajaram V., Kumaratharan N. (2021). Multi-hop optimized routing algorithm and load balanced fuzzy clustering in wireless sensor networks. J. Ambient. Intell. Humaniz. Comput..

